# The Effect of Three Gums on the Retrogradation of Indica Rice Starch

**DOI:** 10.3390/nu4060425

**Published:** 2012-05-29

**Authors:** Rukun Song, Min Huang, Bin Li, Bin Zhou

**Affiliations:** 1 College of Food Science and Technology, Huazhong Agricultural University, Wuhan 430070, China; Email: rukunsong@163.com (R.S.); 478709796@qq.com (M.H.); 790532203@qq.com (B.Z.); 2 Key Laboratory of Environment Correlative Dietology, Huazhong Agricultural University, Ministry of Education, Wuhan 430070, China

**Keywords:** indica rice starch, retrograded starch, konjac glucomannan, carrageenan, gellan

## Abstract

Retrograded starch (RS_3_) was produced from indica rice starch with three kinds of gums (konjac glucomannan, KGM; carrageenan, CA, USA; and gellan, GA, USA) by autoclaving, respectively, and the effect of the gums on the retrogradation behavior of starch was estimated. The influences of polysaccharide concentration, sodium chloride concentration, autoclaving time, refrigerated time, and pH value on RS_3_ formation were discussed. Except for sodium chloride’s persistent restraint on RS_3_, the others all forced RS_3_ yields higher at first, but lowered it after the peak value. The influencing sequence of these impact factors was: sodium chloride concentration > polysaccharide concentration > autoclaving time > refrigerated time > pH value. The results also proved that in the three gums, KGM plays the most significant role in RS_3_ changing. It was concluded that the incorporation of each of these three gums into starch, especially KGM, results in an increase or decrease of RS_3_ under different conditions. This phenomenon could be taken into consideration when developing starchy food with appropriate amount of RS_3_.

## 1. Introduction

Resistant starch (RS) is the sum of starch and the products of starch degradation not absorbed in the small intestine of healthy individuals [1]. RS is reported to possess physiological effects similar to those of dietary fiber, and the physiological effects associated with RS mainly include reducing plasma glucose and insulin levels, increasing fecal bulk, and short-chain fatty acid (SCFA) production through fermentation in the large intestine [2]. It is a promising food additive because of these excellent physiological functions listed above.

The RS is classified into four different types, named RS_1–4_ [3]. RS_3_, retrograded starch, is of particular interest, because of its thermal stability [4,5]. This allows it to be stable in most normal cooking operations, and enables its use as an ingredient in a wide variety of conventional foods [5].

RS_3_ is derived from natural starch during processing. On the one hand, it is a potential food additive, considering the variety of physiological function it possesses. On the other hand, starch retrogradation plays an important role in the processing technology, quality, taste and shelf life of starchy food, in most of which RS_3_ is unwanted. Thus, it is essential to explore both the impact factors and ways to restrain or boost retrogradation.

Starch is comprised of two molecular types: amylose, the straight chain polyglucan, composed of approximately 1000, α-D-(1→4) linked glucoses; and amylopectin, the branched glucan, composed of approximately 4000 glucose units with branches occurring as α-D-(1→6) linkages [5].

In the formation of RS_3_, the starch granule is completely hydrated. Amylose leaches from the granules into solution as a random coil polymer. Upon cooling, the polymer chains begin to re-associate as double helices, stabilized by hydrogen bonds [5,6]. The individual strands in the helix contain six glucose units per turn in a 20.8 Å repeat. Upon further retrogradation the double helices pack in a hexagonal unit cell [5]. 

Starch gels are considered as composites containing gelatinized granules embedded in an amylose matrix. The starch retrogradation can be divided into two steps, the short-term and the long-term retrogradation. The short-term development of gel structure and crystallinity in starch gels is found to be dominated by irreversible (*T* < 100 °C) gelation and crystallization within the amylose matrix. Long-term increases in the modulus of starch gels are linked to a reversible crystallization, involving amylopectin within the granules on storage [7,8,9,10].

In general, crystallization consists of three steps [11,12,13,14]: (1) nucleation, *i.e*., formation of critical nuclei; (2) propagation, *i.e*., growth of crystals from the nuclei formed; and (3) maturation, *i.e*., crystal perfection or continuing slow growth. The extent to which these processes occur is clearly dependent on the temperature of the crystals (*T*_m_); it increases with increasing extent of undercooling (*T*_m_–*T*) or decreasing temperature. At temperatures below the glass transition temperature (*T*_g_), the nucleation rate is negligible; the system is “frozen”. The propagation rate is 0 at *T* < *T*_g_ because diffusion does not occur at such temperatures. At high temperatures, diffusion increases and so does the rate of propagation. At temperatures above *T*_m_, the propagation rate is also 0. The maturation rate is dependent on temperature in a way similar to that of the propagation rate [11]. The overall crystallization rate depends mainly on the nucleation and propagation rates [12].

It could be concluded from the theory of crystallization discussed above that placing starch paste at low temperature first and raising the temperature over *T*_g_ later might facilitate the RS_3_ formation. The promotion of temperature cycle on RS_3_ formation has been studied by some researchers [14]. 

The rate and extent of retrogradation is dependent on both starch botanical sources and treating conditions [15]. It is known that the food system is very complicated. There are many ingredients coexisting with starch that would influence the starch retrogradation behavior. 

In the food industry, there are several kinds of hydrocolloids used to change the structural organization and rheological properties of starchy food [16]. The hydrocolloids are divided into two types, according to their molecular structures: Type A, double helical structure, such as carrageenan and xanthan gum, and Type B, like konjac gum, in which the molecular chain can easily bend, stretch and coil [17].

Most works on starch retrogradation are conducted on starch molecule structure, amylose/amylopetine ratio, incubation time and temperature. Limited reports on starch-gum retrogradation behavior have been published [18,19,20,21,22,23]. Since starch and varieties of hydrocolloids co-exist in processed food, the interaction between them affects the food texture significantly. The objective of this study was to investigate the effect of three hydrocolloids on the retrogradation behavior of indica starch gel under different conditions. 

## 2. Materials and Methods

### 2.1. Materials

Zhongyou 903 (ZY903, no waxy indica) from the first crop of 2005 was obtained from Jingzhou District Agriculture Improvement Station, Hubei, China. Rice samples were dehulled, polished and ground in order to obtain rice flour. The rice flour was treated in the same method of a former research [16] to obtain starch, and stored in a plastic jar at room temperature until used. Konjac glucomannan was extracted and purified from the tuber of *Amorphophallus konjac* [16]. 

Highacyl gellan (H) KelcogelLT100 was provided by CP Kelco Co. (Atlanta, GA, USA). K-carrageenan (BR) and calcium chloride (AR) were purchased from Wuhan Tianyuan biomaterial Co. (Wuhan, China). Alpha-Amylase, pepsase and dried glucamylase were purchased from Sigmae-Aldrich Chemical Co. (St. Louise, MO, USA). HCl (Xinyang chemical Co.), NaOH, KOH (Tianjin Teruijin Chemical Co.), NaCl (Shanghai Sihewei Chemical Co.), Na_2_HPO_4_(AR), NaH_2_PO_4_(AR) from Sinopharm Chemical Reagent Co. (Shanghai, China). They were all used without further purification.

### 2.2. Preparation of Retrograded Starch by Autoclaving

Starch solution was prepared by mixing starch with deionized water at a concentration of 2% (w/w) by mechanical stirring. The solution was pre-gelatinized at 100 °C for 10 min by hot bathing. Then, a different amount of gum as well as NaCl was added to the solution, and the pH value was adjusted by 0.1 M HCl. Afterwards, the mixture was autoclaved (121 °C, 0.115 MPa) for a different time, and cooled to room temperature before being stored at 4 °C for a number of hours. After that, the gel was left at room temperature for 6 h, dried at 80 °C for 18 h, smashed, and sifted through a 63 μm screen.

### 2.3. Retrograded Starch Content

The resistant starch content was measured using the procedure of Goñi *et al*. [24]. The method comprises the following steps: removal of protein with pepsin (Sigma A-7000, 40 °C, 1 h, pH 1.5), incubation with a-amylase (Sigma A-3176, 37 °C, 16 h) to hydrolyze digestible starch, treatment of precipitates with 2 M KOH to solubilize RS, incubation with amyloglucosidase (Sigma A-3514, 60 °C, 45 min, pH 4.75), and determination of glucose, using the glucose oxidase assay. RS was calculated as glucose × 0.9 [25].

## 3. Results

Starch was mixed with water, via high temperature treatment: the starch granule was completely hydrated. Amylose leached from the granules into solution. Following processing, the polymer chains began to re-associate, stabilized by hydrogen bonds, and RS_3_ was formed.

### 3.1. Effect of Concentration of Polysaccharides on RS_3_ Formation

**Figure 1:**
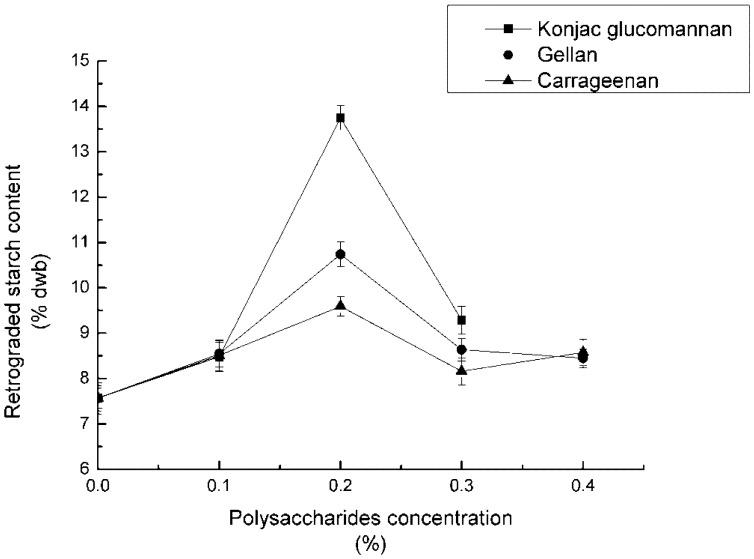
Effect of concentration of polysaccharides on RS_3_ formation.

The concentrations of the three polysaccharides were set at five different levels (0%, 0.1%, 0.2%, 0.3%, 0.4%), respectively. The mixed samples were adjusted to pH 7, added 10% NaCl, autoclaved for 60 min, and refrigerated for 12 h. Every experiment was repeated three times. After the reaction, RS_3_ content was counted and recorded in Figure 1. 

### 3.2. Effect of Concentration of Sodium Chloride on RS_3_ Formation

**Figure 2:**
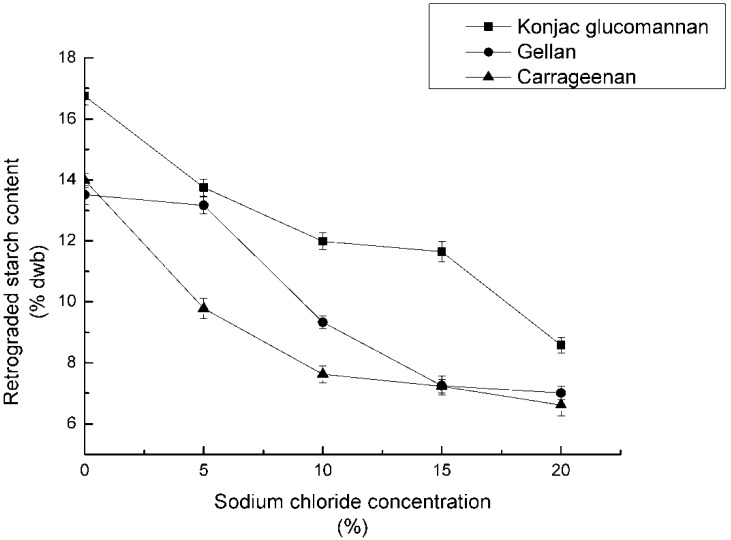
Effect of concentration of sodium chloride on RS_3_ formation.

The concentrations of the three polysaccharides were set at 0.2%. The mixed samples were adjusted to pH 7, and 0%, 5%, 10%, 15% and 20% of NaCl were added respectively, autoclaved for 60 min, and refrigerated for 12 h. After the reaction, RS_3_ content was counted and recorded in Figure 2.

### 3.3. Effect of PH on RS_3_ Formation

**Figure 3:**
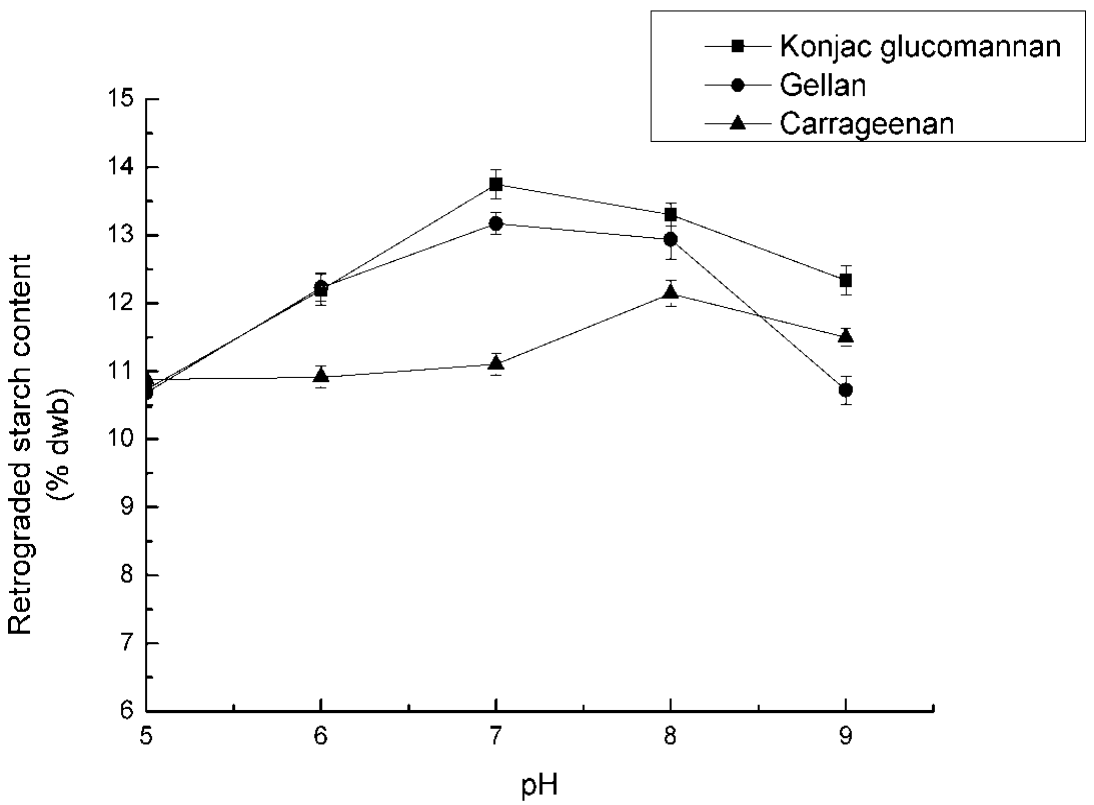
Effect of pH on RS_3_ formation.

The concentrations of the three polysaccharides were set at 0.2%. The mixed samples were adjusted to pH 5, 6, 7, 8, 9 respectively; 10% of NaCl was added, autoclaved for 60 min, and refrigerated for 12 h. After the reaction, RS_3_ content was counted and recorded in Figure 3. 

### 3.4. Effect of Autoclaving Time on RS_3_ Formation

**Figure 4:**
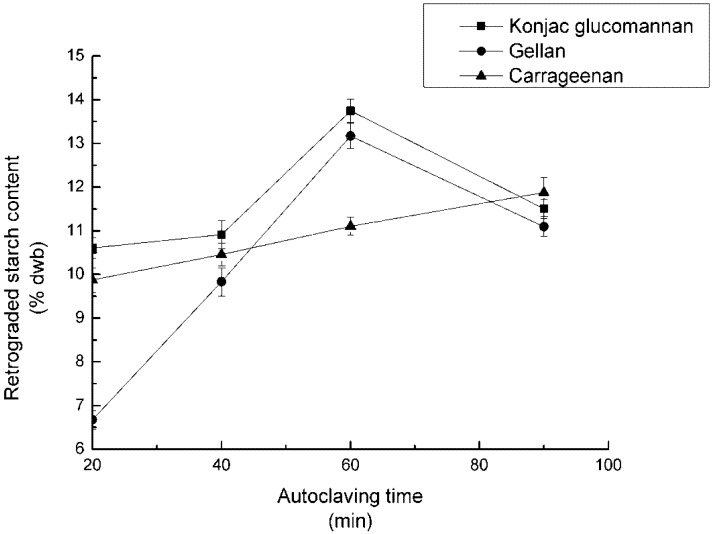
Effect of autoclaving time on RS_3_ formation.

The concentrations of the three polysaccharides were set at 0.2%. The mixed samples were adjusted to pH 7, 10% of NaCl was added, autoclaved for 20, 40, 60, 90 min respectively, and refrigerated for 12 h. After the reaction, RS_3_ content was counted and recorded in Figure 4. 

### 3.5. Effect of Refrigerated Time on RS_3_ Formation

**Figure 5:**
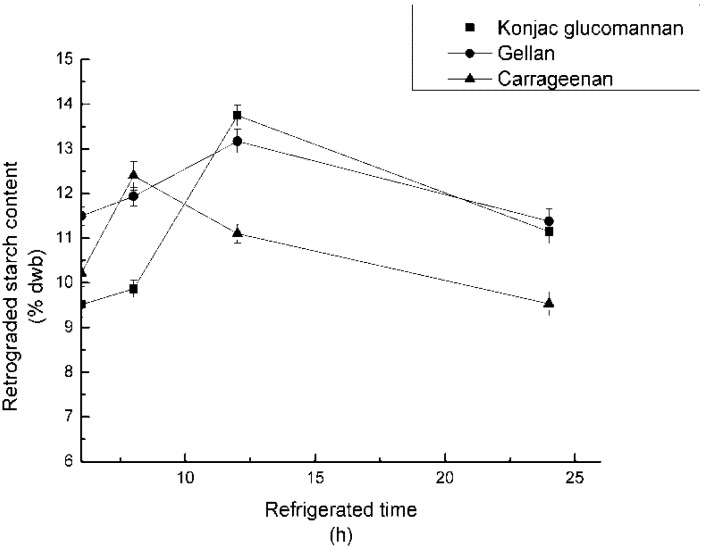
Effect of refrigerated time on RS_3_ formation.

The concentrations of the three polysaccharides were set at 0.2%. The mixed samples were adjusted to pH 7, and 10% of NaCl was added, autoclaved for 60 min, and refrigerated for 6, 8, 12 h, respectively. After the reaction, RS_3_ content was counted and recorded in Figure 5. 

## 4. Discussion

### 4.1. Effect of Concentration of Polysaccharides on RS_3_ Formation

The RS_3_ formation was dependent on polysaccharide concentration. The RS_3_ content kept increasing with the gum concentration rising from 0% to 0.2%, but at higher concentrations, a decrease was observed. This trend indicated that with the increasing concentration of polysaccharides from 0% to 0.2%, the reaction between starch and gum facilitated RS_3_ formation, however, when the concentration was over 0.2%, the solution viscosity was too high for RS formation. 

It could be observed from Figure 1 that the effects of the three gums on RS_3_ formation were different. For concentrations of polysaccharides lower than 0.1%, the RS_3_ content did not change remarkably among the three polysaccharides, but all increased slightly. RS_3_ content with the presence of KGM increased more significantly compared with the other two samples, while the polysaccharide concentrations were between 0.1% and 0.2%. The addition of CA had the smallest effect on RS_3_ formation. Furthermore, there was a sharp decrease of RS_3_ content with the presence of KGM within polysaccharide concentration from 0.2% to 0.3%, while a slow decrease with the presence of the other two gums. When the concentration of polysaccharides was more than 0.3%, no remarkable change of RS_3_ content was observed.

The interaction between polymers is a very complex process, which could be influenced by a range of factors. With other conditions (temperature, pH, *etc*.) being kept equal, the different characteristics of the gums co-existing with the starch could be employed to explain the results. According to the former research of the same team [16], RS_3_ content was bound up with the properties of the starch-gum gel. During storage, the lower the viscosity of the gel, the more violent was the molecular motion. Violent molecular motion was in favor of the formation of a more compact double helix structure. Contrariwise, high viscosity blocked the molecular motion of the gel, and resulted in less crystal nucleus. As has been found with increasing KGM concentration, the adhesiveness of the mixed gel did not change significantly [16]; thus, the presence of KGM boosts the leaching of amylose during gelatinization. In addition, the molecular motion of amylose facilitates the formation of a crystal nucleus during storage of the starch-gum mixture. 

### 4.2. Effect of Concentration of Sodium Chloride on RS_3_ Formation

The RS_3_ content showed a steady decrease as the level of sodium chloride in the reaction mixture increased. The maximum RS_3_ content was observed at the level of 0% of added sodium chloride. Results of these experiments indicated that the addition of sodium chloride impeded the hydrogen bond formation between the starch molecules, resulting in less RS_3_ content.

When salt was added to the starch-gum solution, the inter-molecular associations between starch molecules, starch and salt, as well as salt ions, changes the flexibility of the starch chains. In addition, the interaction between salt and water molecules restricts the molecular motion of the starch, resulting in less recombination of the amylose. There are two possible hypotheses, as follows:

On the one hand, the NaCl added to the samples ionizes after moisture treatment. Because of the presence of Na^+^ and Cl^−^, water molecules lose the ability to form a tetrahedron structure, which leads to the decrease of interactions between water and starch. This phenomenon has also been found in the research of Viturawong [26]. In this regard, it was positive for RS_3_ formation.

On the other hand, the electrostatic force of Na^+^ reduces the mutual exclusion of glue molecules. 

The formation of gel depends on the balance of repellant and attraction. It was shown that the RS_3_ decrease was the result of comprehensive action of the two effects above, and the later one was stronger. 

### 4.3. Effect of pH on RS_3_ Formation

It could be perceived that the pH played a less important role in the formation of RS_3_. From Figure 3, the optimal pH was 7 for KGM and GA addition, and pH values that were too high or too low pH decreased the RS_3_ output. It could be attributed to the degradation of the starch molecules at low pH, which would inhibit the formation of crystals. While at high pH, the presence of hydroxyl anions would be unfavorable for hydrogen bond formation. Moreover, there was no electrical repulsion under neutral conditions.

With the addition of CA, the RS_3_ content reached a peak at pH 8, which was different from the other two gums. KGM still exhibited the best facilitation on RS_3_ formation when the concentrations of the three polysaccharides were all set at 0.2%.

### 4.4. Effect of Autoclaving Time on RS_3_ Formation

With respect to the autoclaving time, there were two modes of change in RS_3_ content. Samples with KGM or GA displayed the first one. It showed that by extending autoclave time, the RS_3_ content increased first, and decreased over the peak value, which occurred at 60 min. While in the samples with CA, the RS_3_ increased steadily extending autoclaving time. 

The conclusion of Figure 4 was that prolonging the autoclaving time in a certain range is positive for RS_3_ formation. Long time autoclaving led to a more complete starch gelatinization, and the release of amylose molecules. Consequently, the polymer chains re-associated more easily. However, the starch molecules break down into small ones, which are unfavorable for the formation of hydrogen bonds.

Since the molecular types of the gums were different [27], they interacted with starch molecules in different ways. At the early stage of autoclaving, starch molecules were packaged up by gums. It was difficult for the starch chains to band together by hydrogen bonds. The interaction between starch and KGM was not strong enough to repress the RS_3_ synthesization under high pressure, due to the gum’s B molecular structure [17], and it was the same to sample with GA addition. As for CA, the interaction between it and starch chains was so strong that RS_3_ content cannot increase rapidly even when the mixture had been autoclaved for 90 min.

### 4.5. Effect of Refrigerated Time on RS_3_ Formation

It can be learnt from Figure 5 that samples with KGM or GA addition could yield the most RS_3_ after 12 h refrigeration at 4 °C. The RS_3_ increased rapidly from 8 h to 12 h, and then decreased slowly in the presence of KGM. Meanwhile, the sample with CA addition showed a different tendency in RS_3_ formation. During refrigeration, starch co-existed with CA, displaying signs of minimal retrogradation. Compared with the other two groups, the change of RS_3_ with GA was moderate.

What interested the researchers most was that after a certain time of refrigeration, the RS_3_ decreased in all the three experiments, which indicated that it might be possible to control the RS_3_ content at a desired amount by adding food gums to the frozen food. However, it was difficult to explain RS_3_ degradation over the peak. A possible explanation was that the starch-gum mixture formed reversible crystals, which might be linked with the long-term retrogradation mentioned in the introduction [7,8,9,10]. More analytic methods need to be employed in the future to explain this confusing phenomenon, 

## 5. Conclusions

The present study shows that the five factors which have been investigated above can influence the RS_3_ yield of starch-gum mixtures. Further data analysis indicates that the scale of the influence is (from high to low): sodium chloride concentration, polysaccharide concentration, autoclaving time, refrigeration time, and pH value. In addition, different gums display various effects on RS_3_ formation. In general, the KGM-starch mixture produced the maximum RS_3_, comparing with the other two gums under the same experimental conditions. The RS_3_ produced with each of the three gums is reversible, indicating that the properties might be different from that produced without the gums.

From the synthesization of this research and the other researchers [20,21,22,23], it can be concluded that the addition of food gums to starch paste exerts either a positive or negative influence on starch retrogradation. It is determined by the kind as well as the concentration of polysaccharides, and the processing conditions are also important. In other words, many more factors should be taken into consideration in facilitating or weakening starch aging. 

The present data demonstrate that it is possible to add gums to starchy food formulations to increase or decrease RS_3_ formation. With an increasing demand for starchy foods with different qualities, it is likely that these functional ingredients will become more important to the starch food industry. However, interactions between starch and gums during autoclaving are not well understood. The way in which these gums affect the starch retrogradation requires further investigation.
